# Food insecurity in college students: a qualitative exploration of the multiple domains

**DOI:** 10.3389/fpubh.2026.1872467

**Published:** 2026-07-03

**Authors:** Alyssa Anderson, Jennifer King, Quinn Parker, Cassandra Johnson

**Affiliations:** 1 School of Health Sciences, Kent State University, MACC Annex, Kent, OH, United States; 2 College of Public Health, Kent State University, Lowry Hall, Kent, OH, United States; 3 School of Family and Consumer Sciences, Texas State University, San Marcos, TX, United States

**Keywords:** basic needs, campus food security, food access, food security, higher education

## Abstract

To help address student basic needs, universities may benefit from screening students using a tool that captures multiple food insecurity domains. The purpose of this study is to explore college students' experiences related to the various domains of food insecurity (quantitative, qualitative, social, and psychological). This qualitative research was conducted at a Midwestern public research university through semi-structured interviews with embedded surveys that measured food insecurity. 23 undergraduate and graduate students participated. Three major themes emerged from the interviews. First, students were consistently spending time and energy navigating ways to secure their next meal, which was reflected through experiences in all four domains (quantitative, qualitative, social, and psychological). Second, differences in resource restrictions and accessibility influenced the consistency and amount of food accessible to student which impacted quantity of food received, held psychological consequences for students, and affected decisions they made socially. Finally, students felt the quality of food available to them negatively impacted their health both short- and long-term which was related to negative psychological consequences such as added stress and anxiety. Widely used tools such as the USDA 10-item screener mainly capture the quantity of food available and more extreme forms of food insecurity. However, as seen in the interviews, many students may still be experiencing varying levels of food insecurity and barriers (i.e. social stigma) related to the qualitative, social, and psychological domains.

## 1 Introduction

Food insecurity is a major issue faced by college students (1). The Hope Center 2023–2024 Student Basic Needs Survey Report found that 41% of students across the United States experience food insecurity (2). Longterm, food insecurity can impact students' academic success (3, 4), physical health (3–5), and mental health (2, 6).

Scholars have reported on the negative significant impact of food insecurity on academic performance (3, 4) and success related to graduation (4). Among a sample of 508 undergraduate students at a midwestern university, there was an association between food insecurity and lower grade point average (GPA) scores (7). Similarly, a study of 354 students in the western U.S. found that students with GPAs higher than 3.1 were 60% less likely to be food insecure (8).

In addition to its influence on academic performance, food insecurity is also connected to adverse health outcomes (3). Students reporting food insecurity are more likely to have a lower quality diet, greater perceived barriers to cooking and food preparation, and a higher body mass index (BMI) (5). Similarly, risk of obesity, worse health outcomes, and general reports of poor health have been associated with increased food insecurity among college students (8, 9).

In relation to student mental health, 44% of all students experience clinical symptoms of anxiety and depression (2). Previous research has found an increased prevalence of psychological distress and average to very poor mental health status in students with food insecurity when compared to food secure students (6). Feelings of depression and anxiety associated with food insecurity also appear to be increased in this population, while scores related to feelings of hope were decreased (3).

Qualitative studies have provided in-depth insight into students' experiences with food insecurity. For instance, semi-structured interviews across a midwestern state found that students experiencing food insecurity spent a great amount of mental energy on securing food which increased their feelings of anxiety and stress (10). In addition to this, the stigma of food insecurity led to them trying to hide their hunger from others (10). Common coping strategies for food insecurity include attending events with free food, buying inexpensive foods that tend to be less healthy, and stretching food to last longer (11).

The college student population consists of diverse social identities and perspectives. Pervious research has demonstrated that some students are more vulnerable to experiencing food insecurity due to these identities. Students with a disability have been shown to experience nearly twice as high of rates of food insecurity in comparison to students without a disability (12, 13). Additionally, students that identify as LGBTQ (14), students of color (12, 15), and post-traditional undergraduates (16) have been found to be at an increased risk for facing food insecurity. Post-traditional undergraduate students' characteristics vary by study and may include caregiving responsibilities, being 25 years of age or older, military experience, and delayed enrollment into college by at least one year (16–18). Likewise, international students face unique social and financial barriers that impact food access (19).

When reporting on college food insecurity, the two-item screener version of the U.S. Adult Food Security Survey Module (AFSSM) (20) is the preferred tool to use amongst college students because of its relative accuracy and its widely adapted use in various settings (21). However, researchers have noted the AFSSM has not been formally validated for use specifically within the college student population, which may lead to the misclassification of students' food insecurity level (21, 22). This limitation also impacts other populations. Recent research has focused on developing more holistic measurement tools to address this issue. The Center for Nutrition and Health Impact has been leading the development and use of complimentary measurement tools that capture the additional dimensions of availability, utilization, and stability among a general population of U.S. adults (23, 24). Another complimentary tool, the Four Domain Food Insecurity Scale (4D-FIS), measures food access based on quantitative, qualitative, social, and psychological domains (25). The inclusion of the social and psychological domains of food insecurity is relevant for understanding food insecurity in relation to mental health and social stigma.

These innovative methods and frameworks have not yet been explored within the college student population. While these tools are valuable for quantitative data gathering, they also provide the opportunity to serve as a framework for qualitative studies. This qualitative exploratory study focuses on using the 4D-FIS tool (25) as a framework for semi-structured interviews to gain a deeper and holistic understanding of how students' experiences with food access relate to these domains. Specifically, we aim to answer the following research question: How do college students experience food insecurity in relation to the qualitative, quantitative, social, and psychological domains? These findings can expand the understanding of food insecurity among college students of various social identities, be used to inform the development and testing of future food security measurement tools, and be leveraged for campus food security initiatives.

## 2 Methods

### 2.1 Design

This study is part of a larger project on campus food security at a multi-campus, public, four-year research university in the Midwest region of the United States. The campuses are in suburban and rural communities and are within commuting distance of major cities. The goal of this project is to explore students' experiences with food insecurity and how the environment influences these experiences through the collection of both quantitative and qualitative data. Ethical approval for the study was obtained from the Office of Research Compliance at Kent State University.

The study team recruited from participants who completed a survey related to student food insecurity in Spring 2024. Originally, survey participants were recruited through an email that went out to 28,433 graduate and undergraduate students enrolled across the university campuses. Participants that expressed interest (*n* = 335) in a future interview received an email invitation. Invitations to participate with reminders were emailed three times throughout the semester in fall 2025, with the first being sent 1 month after classes started.

Eligible participants were 18 years of age or older and currently enrolled in classes. Both food secure and food insecure students were invited to participate due to the nature of quantitative measurement tools tending to capture only the more extreme cases of food insecurity (21, 22). The study team completed screening by ensuring that those interested in participating met the inclusion criteria. All volunteers that met the inclusion criteria were given the opportunity to schedule an in-person or virtual interview through Microsoft Teams. Informed consent was obtained from participants and those who completed the interviews were offered a gift card valued at $20.

### 2.2 Theoretical and conceptual framework

This study utilized the 4D-FIS measure as the conceptual framework (25). This framework is based on research that focusses on the multiple dimensions of food access and how they relate to food insecurity (26–29). Radimer et al., defined food insecurity as a “managed process”, highlighting how food insecure individuals often implement coping mechanisms related to food access that impact their mental and physical health often before quantitative deficiencies in food are measurable (26). This framework, displayed in Table 1, captures food security through four main domains: quantitative, qualitative, psychological, and social (25).

**Table 1:** Theoretical framework: four domains of food insecurity.

Domain	Description
Quantity	Insufficient quantity of food and/or depletion of food.
Quality	Inadequate quality of food and/or unsuitable food.
Psychological	Uncertainty, worry about food, lack of choice, and/or feeling deprived.
Social	Social unacceptability and/or disrupted eating patterns.

Adapted from Johnson et al. (25).

### 2.3 Data collection

Data collection relied on semi-structured interviews with embedded surveys. Each participant completed one interview. For measuring food security level, participants responded to two surveys: the 2-item screener version of the 10-item U.S. AFSSM (20) and the 4D-FIS (25) to assess multiple domains of food access. The 2-item screener version of the 10-item U.S. AFSSM was chosen because it is a commonly used measurement in food security studies (21). The 4D-FIS was used in addition to this because this measure of food security aligns with the theoretical framework of the research study.

The 10-item AFSSM measures food insecurity level based on the number of affirmative responses and categorizes participants into four groups: high food security (0 points), marginal (1–2 points), low (3–5 points), and very low (6–10 points) (20). The 2-item screener was used in conjunction with the survey to help reduce survey burden in participants who had high food security; those that responded to the screener with two affirmative responses did not complete the remaining 10 questions (20).

While the 10-item AFSSM questions focus mainly on capturing the quantity of food available, the 4D-FIS was designed to represent the four domains (quantity, quality, social, and psychological) more equally (25). This tool measures food insecurity based on the last 30 days and groups food insecurity into three categories: food secure, mildly food secure, and severely food insecure. Details on the scoring of this tool can be found in the original article (25).

A team of three interviewers conducted semi-structure interviews. Interviews were designed to last about 60 min. The guide included open-ended questions and probes. Interviews started with an introduction and ice breaker followed by the first food security measurement tool (AFSSM or 4D-FIS). Next, participants were asked open-ended questions about their experiences and knowledge of food assistance resources followed by the second food security measurement tool (AFSSM or 4D-FIS). Finally, participants completed a set of closing questions and open-ended questions related to demographic information on race/ethnicity and gender. The study team recorded and transcribed interviews. Personal identifying information was removed during transcription and participants were assigned pseudonyms to maintain anonymity.

### 2.4 Data analysis

Interview lengths ranged from 14 min to 56 min with a total of 685 min of recorded interview time. The scores were calculated for the 4D-FIS (25) and 10-item AFSSM (20) for each of the 23 interviewees. The wide range in interview times generally coincided with food security levels; those students experiencing higher food security tended to have shorter interviews compared to those students facing lowers rates of food security. Descriptive statistics on food security level for both tools were run using IBM Statistical Package for the Social Sciences (SPSS) software (version 29).

Coding was completed independently by three coders using NVivo14. Qualitative data analysis included deductive and inductive coding. Initially, two coders deductively coded all transcripts using the four dimensions of the 4D-FIS framework as *a priori* codes. These four codes were then discussed between coders and inductively coded with properties as displayed in Table 2 (30). Throughout the process, the coders met frequently to compare, discuss, and revisit coding results and form the codebook. After the codebook was completed, a third coder coded half of the transcripts. To reduce potential for bias, investigator triangulation was completed as all three reviewers compared notes to ensure agreement and consistency in coding and the related properties observed (30). Memos, codes, and final themes were discussed and agreed upon by the research team.

**Table 2:** The four domains of food security: each domain's properties and property description for coding.

Domain	Property	Property description
Quantity	Food provided by resources	Insufficient amount of food provided by food assistance resources.
	Loans and credit	Use of loans and credit to obtain sufficient food.
	Smaller and less frequent meals	Skipping or stretching meals due to insufficient amount of food.
	Food accessibility	Insufficient amount of food due to lack of transportation and locations of food resources.
Quality	Perception of food safety	Views of food being unsuitable for consumption due to safety and health concerns.
	Time constraints	Consuming lower quality foods due to time constraints.
	Food costs	Consuming lower quality foods due food costs.
	Resource offerings	Consuming lower quality foods due food assistance resource offerings.
Psychological	Amount for immediate consumption	Uncertainty and worry about having enough food at that moment.
	Planning and preparation	Uncertainty, worry, and feeling a lack of choice related to planning and preparing meals.
	Family role and responsibility	Uncertainty, worry, and feeling a lack of choice related to role within the family.
	Resource usage	Uncertainty and worry related to using a food assistance resource.
Social	Stigma	Experiencing feelings of social unacceptability related to accessing food.
	Life transitions	Disrupted eating patterns due to social changes in life.
	Culturally appropriate foods	Disrupted eating patterns due to lack of culturally accessible foods.

### 2.5 Rigor

Strategies used for promoting reliability and validity include, the pre-testing of the interview guide, having members on the research team trained in qualitative research, coding based on a relevant theoretical framework, peer debriefing, and member checking. To promote trustworthiness of data, interviewees were given 2 weeks to check their transcriptions for accuracy and provide additional information for clarity if needed (31).

## 3 Findings

Twenty-three interviews were completed. Participants' demographic characteristics are shown in Table 3. The 10-item AFSSM classified 39% of the interviewees as food insecure (low and very low food security categories). In comparison, the 4D-FIS found 48% to be severely food insecure.

**Table 3:** Participant characteristics.

Characteristics	*n* (*n* = 23)	%
Gender		
Female	15	65
Male	8	35
Self-reported race/ethnicity		
Asian	2	9
Bangladeshi	1	4
Black/African American	3	13
Hispanic/Latino	1	4
South Asian	1	4
White	14	61
Student academic classification		
Graduate: doctoral	6	26
Graduate: master's	6	26
Undergraduate: sophomore	5	22
Undergraduate: junior	5	22
Undergraduate: senior	2	9

**Figure 1:**
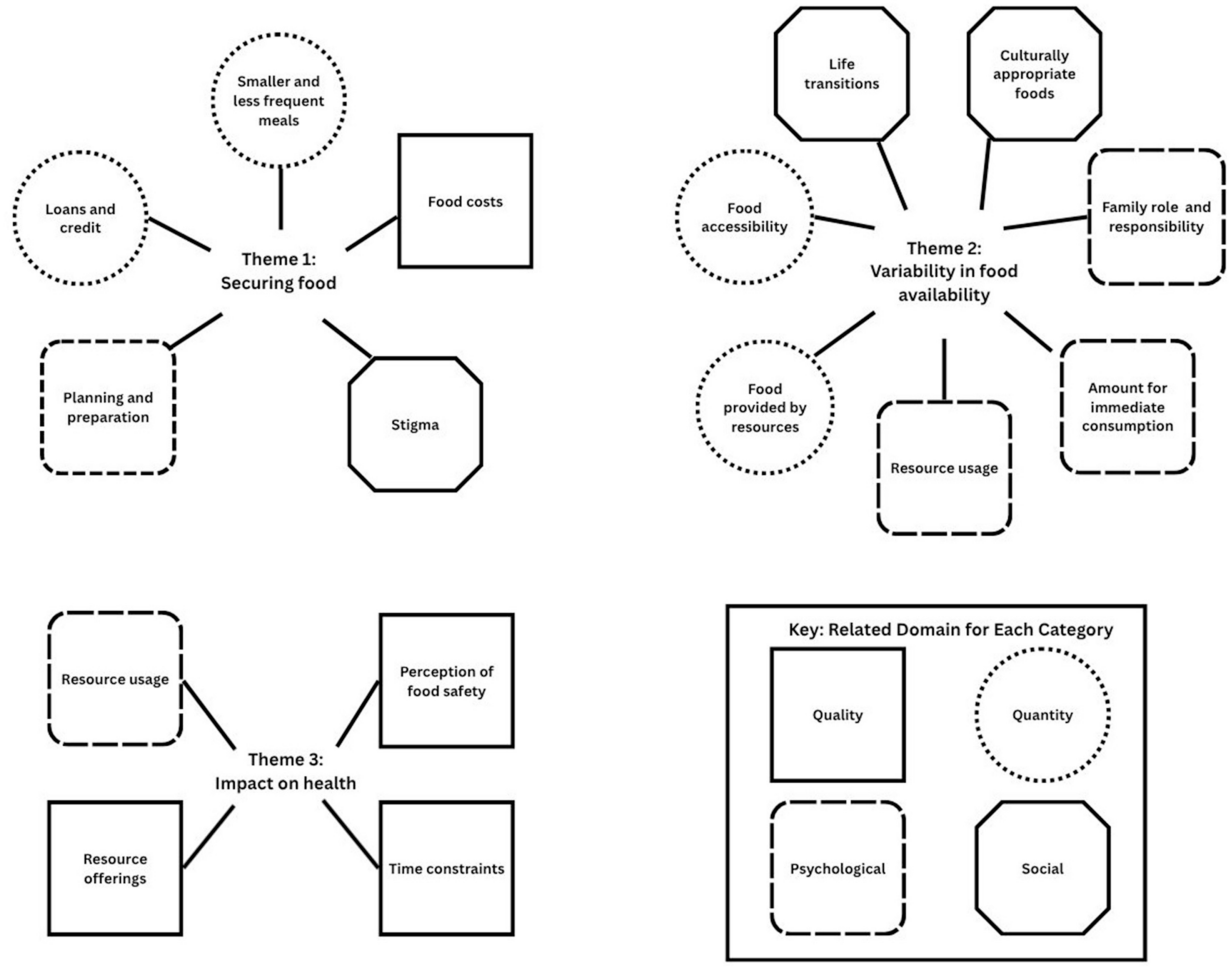
Illustration of how the four domains and their specific properties connect to the main themes.

Three main themes emerged from the interviews that capture students' experiences with food insecurity as a “managed process” through the framework of the different domains. The themes of student experiences with food insecurity are as follows: navigating the securing of sufficient food, the variability in food availability from resources, and the short and long-term impacts of food choices on health. Within each theme, the different domains (psychological, social, quantity, and quality) and their respective property(s) shape student experiences with food insecurity. Figure 1 highlights the three main themes captured through the 4D-FIS domains (each shape represents a corresponding domain) and the specific properties related to those domains that influenced that experience. Table 4 compliments Figure 1 by providing examples of participant quotes related to each property. Abbreviations that follow participant pseudonyms within the results and findings represent assigned individual 10-item AFSSM (20) categories at the time of the interview and are as follows: very low food security (VLFS), low food security (LFS), marginal food security (MFS), and high food security (HFS).

**Table 4:** The four domains of food security: related properties of *a priori* coding and example quotes of each property.

Domain	Property	Example
Quantity	Food provided by resources	*[Related to food pantry] You get just like a small bag of food after you wait 30-45 minutes. I mean, it's better than nothing, but it's still, you know, after waiting all the time, you really don't leave with very much. (Leah, VLFS)* *[Related to SNAP] They actually count my Social Security as income, and so I get $23 a month...that's not enough to pay for a day of food for me. (Nancy, LFS)*
	Loans and credit	*The other thing that I want to say is that I do pay for the majority of my food with a credit card...I do not have enough money allocated from my Social Security disability to pay for food. (Nancy, LFS)* *I don't pay for school with my loans. I pay for my living expenses with my loans because I do not earn enough. (Rebecca, HFS)*
	Smaller and less frequent meals	*Most of the time throughout the day I'm not as hungry, but it's mostly towards dinner time where I may like cut like a meal short or something like that, and then I normally wait till breakfast to eat again. (Takeya, LFS)* *There's definitely times I have to think through um with how I um spend my money. Definitely like a like a couple times throughout the week I have to like look at my bank account and be like ok well, we might have to just go straight home today. Or um instead of having lunch now, we might have to have lunch a couple hours before when I'm home. (Jasmine, MFS)*
	Food accessibility	*Sometimes um I think maybe for the international students... I know most of them have the money for food, but they cannot get access to the grocery stores they want because they don't drive. (Jun, LFS)* *I feel like sometimes these services don't think about that. Okay, we have all this food, but how are these people supposed to get it right? Like public transportation is not that easy here. (Priyanka, HFS)*
Quality	Perception of food safety	*So our campus has, like, these grocery stores where they have these prepackaged meals that many students get with their meal plan. And so once those prepackaged meals become....umm...A little bit umm once their expiration date gets closer, they usually give those meals to the pantry, I believe. And then we get, like basically like, what's leftover...essentially. (Takeya, LFS)* *I didn't eat balanced meals at the dining hall, if that makes sense…because of my allergy restrictions. (Rebecca, HFS)*
	Time constraints	*Um I'm not sure if this would like help better like understand like some of the answers I give but uh for the most part most of my food like comes from just fast food itself. (Jasmine, MFS)* *I ate uh less protein because they are difficult to prepare compared to like vegetables. (Jun, LFS)*
	Food costs	*Um you know, so just just making different choices and eating similar foods over and over again because they're more affordable than what we used to eat. Um that's definitely been a change. (Elizabeth, MFS)* *[when talking about not eating foods they thought were important] I don't know, meat's expensive, but like, I place a little bit of emphasis on that. (Lucas, HFS)*
	Resource offerings	*No, I think the only thing that I was frustrated with this like pantry that I went there twice, just the type of food they give and then they talk about like then you see all these billboards that “oh feed your child healthy food”, you know, “give them healthy snacks”. But then they were giving out those like not healthy snacks, right? (Priyanka, HFS)* *[when discussing a food pantry on campus] The quality of the products can sometimes be inconsistent. (Takeya, LFS)*
Psychological	Amount for immediate consumption	*So at that time I feel a little bit anxious because sometimes I-I see there's no food in my fridge. (Jun, LFS)* *[when discussing feelings of anxiety about getting enough food] I get paid biweekly, so around the time that I don't get paid my paycheck that week. (Kendell, VLFS)*
	Planning and preparation	*I would say like fall/winter seems to be more...more costly or there is more anxiety related to it I think. Maybe because summer is like where you can get away with grilling which is so much cheaper. (Evelyn, MFS)* *I usually...um always plan ahead, like okay, this is what I'm gonna do, this is who I can call. So I may worry for a little bit, but then I just normally just get into action. (Takeya, LFS)*
	Family role and responsibility	*It was a very long line, and my child was small at that time. I didn't think it was worth it my time and effort and all that. (Priyanka, HFS)* *I have a cat and if money's tight, I always prioritize her eating. (Aleena, LFS)*
	Resource usage	*The state government does offer food assistance, but I do know that there's like certain stipulations around that. Whether a college student can qualify, they have to work 20 hours a week and then I believe they have to be under a certain amount of income, which they make it like so low. It is hard for anybody to qualify really, but*... *they also have a website where you could apply online if you didn't wanna call up there 'cause they do have extremely long waiting times. (Leah, VLFS)* *Just ‘cause there's more hoops to it, and it's only one time. Um and dealing with graduate student Senate is like very formal, and it's like a headache in itself (laughter), um trying to figure out if there's like gonna be a deadline or a cutoff. Um so that's like the only one that I've decided not to use. (Aleena, LFS)*
Social	Stigma	*I always get a little bit anxious not because of like needing the services, cause I don't think that there's like anything embarrassing inherently, like everybody needs help at some point. But I think, some people...don't...give a lot of dignity using those services. (Aleena, LFS)* *I mean, I'd probably feel like a little embarrassed, but also I know that like people need help sometimes. So I-I wouldn't feel too bad. I-if anything I might feel like I'm taking it from people that might need it more than I would. (Jane, HFS)*
	Life transitions	*We used to carpool together, but now I have too many kids they won't all fit in her car with us. (Leah, VLFS)* *And to access to food is also different because my current roommate cooks a lot, she enjoys cooking, so we share our meals. Last year we didn't share. We didn't eat together and we have everything separated. (Iryna, HFS)*
	Culturally appropriate foods	*I feel that some of the communities are not thought about when designing meals. As I said, for the Muslims, especially any kinds of meat. They prefer those halal foods, but since it's not available anywhere that I'm aware of they just try to avoid those places. (Ajay, VLFS)* *[when discussing food from a resource] I eat like my cultural food, like my ethnic food, right? So, it was not helpful. I ended up giving out all like everything. (Priyanka, HFS)*

### 3.1 Theme one: securing foods

While most students interviewed were getting an adequate amount food to meet their minimum needs, they lacked access to a sufficient amount of food to meet all their needs and preferences. As a result, many of the students were consistently spending time and energy navigating ways to secure their next meal despite ranging over the different levels of food security measured by the 10-item AFSSM. For example, while some students were able to access meals regularly, it was not without long-term economical consequences due to the use of loans or credit cards to help purchase the food. As shared by Rebecca (HFS), *I have an overarching nuance of I ended up taking out loans and that's what really makes me be able to afford my living expenses*. And, in the more severe cases of food insecurity, skipping meals was also noted by students:

*I do it for dinner I would say 'cause I'm not forcing myself to go throughout the day hungry. Instead, I'm in my dorm and I can sort of like maybe drink water or something like that to sort of distract my mind and then that's when I'm doing homework. So, although I do it for breakfast sometimes, I mostly do it for dinner. (Takeya, LFS)*

There are social and psychological consequences related to this mindset of consistently navigating food access. Socially, stigma often prevents the use of different food related resources because students felt their situation was not “bad enough” to warrant usage. They viewed these resources as a last resort and shared sentiments like this:

*Even though sometimes I may question my own availability to like food, I don't feel as though I'm in a bad enough situation that I would need to take from those facilities and I would rather somebody who is in dire need for those resources to have access to it because I'm capable, more capable of getting the resources I need. (Angela, VLFS)*

Psychologically, this extra work of securing resources takes an emotional toll on students. The anxiety of the future, due to knowing how quickly food security can change, did not go away. As shared by Kendell (VLFS),

*I get really paranoid about using my meal plan because I do have the 200 block.... Because I'm always scared that I will run out of the meal plan, so sometimes I choose not to eat when I feel like I do need to eat*.

Finally, students control their situation by considering economic factors and navigating their choices with this in mind, often resulting in sacrificing the quality of their food. Evelyn (MFS) discussed how this impacted her food choices and stated, *I would say it's more a matter of getting a cheaper version of a food at a grocery store because the better for you/better quality ones are more expensive*.

### 3.2 Theme two: variability in food availability

Variations in resource restrictions and accessibility influence the amount of food available over time. This access can vary by semesters and academic years. Transportation, hours of operation, and availability of culturally relevant foods in grocery stores all affect students' access to enough food. When Ajay (VLFS) was discussing why he stopped using a food pantry off campus, he mentioned how his friends would take him in their vehicles and then added, *those people moved, and now I don't have transportation*. And, while food assistance programs, such as the supplemental nutrition assistance program (SNAP) or campus food pantries, help in securing some food, it is often still not enough to meet students' needs. In some cases, resources run out of food before everyone is seen. As stated by Leah (VLFS), *if you don't get there early enough, they do tend to run out of stuff like the further back in line you are. It's more of a first come, first serve situation*.

When students use resources, this choice is often accompanied by psychological stress and a level of uncertainty. For example, Leah (VLFS) expanded on her past experiences by sharing,

*Well, that one always says it's supposed to start at 12:00, but you get there and you're waiting in line for over an hour. You know, 'cause, they have to let out the people from church 'cause it is held on a Sunday. So, you end up sitting there in your car spending so much on gas waiting for it*.

This shortage of food, whether currently experiencing it or being concerned about the future, has a noted psychological impact on students and comes up in statements like this one from Aleena (LFS) when talking about grocery shopping: *Um a couple times the last month where I've been like, ok...prices are rising, how am I gonna get enough?* In addition to this, students with caregiver roles, whether for children or pets, often had added sources of stress and responsibility around securing food. Evelyn (MFS) described her caregiving experience as:

*I am cooking for a family so some of that is just like it is all really expensive. And, you know, it's not just breakfast, lunch, dinner for me but also it's packing lunches for the kids and things like that*.

Students' social lives are complex and evolving throughout undergraduate and graduate school which can impact their level of food access. Life transitions (i.e., international students moving abroad, forming new peer/community networks, living along on or off campus, and commuting) commonly occur and can result in drastic changes depending on the semester. As Rebecca (HFS) notes when discussing the use of an on-campus food assistance resource, *I used it when I was like a junior to senior undergrad, I used it a lot and it really helped out because it was my first time in an apartment*. Additionally, the recent and ongoing changes related to government food assistance and basic needs programs impacted students' outlook on future food availability. For example, Nancy (LFS) noted how quickly and drastically her situation could change because of this: *All the programs that I depend on to live may be cut*.

Finally, international students often struggled to find consistent access to affordable and culturally relevant foods. Students would discuss having to drive to nearby cities to find familiar foods. Jun (LFS) discussed traveling with his friends almost an hour for some foods:

*For some food I prefer, I cannot find them here easily. So sometimes if I really need them, I need to drive to [city]. But it's not an economical way to always drive to [city]… and those food can be expensive in this country so I may not have them all the time*.

### 3.3 Theme three: impact on health

Participants shared perspectives and experiences about how food access negatively impacted their health both in the short- and long-term. In situations where there was still adequate food to eat, there was still sometimes a high level of concern about the safety of the food. Perceptions of quality and safety of food led to avoidance of certain foods and an increased reliance on shelf stable, processed convenience foods and fast foods.

Particularly among international students, there was a general distrust in the safety of the food at certain grocery stores, campus dining halls, and food assistance resources. Iryna (HFS) shared the following about her experience as an international student and securing food:

*I still don't eat everything because I'm not sure if it's like enough safety to eat...especially in the beginning I wasn't sure like about the quality of food because I was really scared about the health system here*.

Many students also shared some distrust related to the health impacts of offerings from food pantries. Some of these concerns were related to food safety. For instance, Jun (LFS) mentioned that *some of the food there are not that fresh, so sometimes you get a whole box, but when you got home you need to throw away some of them*.

Additional concerns were related to long-term health impacts due to lower quality of items. For instance, with food pantries,

*It will help you...I don't know help you at least to get by the day. But then it's also not making you healthy. It's not healthy choices that they give you. (Priyanka, HFS)*

This then often leads to feelings of stress when having to use these resources as the primary source of food. As Priyanka (HFS) went on about her experience with food pantries she shared,

*They end up not being healthy at the end, you know? Then we put the pressure on them. Oh, you didn't eat healthy. But no, we didn't provide you healthy food. Yeah so, and I mean, yes, those are the resources, but I don't know how I feel about that*.

Even when students purchased their own foods, constraints such as a busy schedule and cost of food, resulted in students being limited to convenience foods that they recognize as less nutritious options. Aleena (LFS) shared that *oftentimes I do have boxed food and canned food as like a backup*.

## 4 Discussion

Based on the 10-item AFSSM, those students who volunteered to participate in interviews faced higher rates of food insecurity (39.1%) compared to the food insecurity rates (28.0%) found in a campus-wide survey within this same setting in Spring 2024 (16). The use of the 4D-FIS framework for coding allowed for us to capture students that “used coping tactics to avoid or delay some components of hunger” (26) which was not always reflect through only the 10-item AFSSM scores (25). These different tactics and strategies, which align with previous research, emerged as students discussed grocery shopping, food assistance resource usage, and the use of loans and credit cards to cover daily living expenses (11). Additionally, because of the ever-changing dynamic of students' lives, focusing on the quantitative domain by primarily measuring the amount of food consumed based in the last 30 days did not always capture the entire experience of students which includes psychological uncertainty, social stigma, and the concerns of food quality.

Experiences related to the social domain highlighted that despite students creatively finding ways to access food, they minimized their experiences of need or didn't feel their situation was extreme enough to receive aid from different food resources. This is of considerable concern as resource use is often stigmatized by social labeling of being unacceptable and “perpetuates self-blame” for one's circumstances (32). Social challenges that were relevant to international students in particular include food accessibility, life transitions, culturally appropriate foods, and perception of food safety. These challenges that emerged from the interview support previous qualitative research findings related to international students' experiences with barriers to food access (19). As college students may hold multiple identities, it is worth noting the potential impact of intersecting stigmatized identities that can deter food and other basic needs assistance resource use.

While students have access to adequate food, the quality domain underlines the importance of considering various short- and long-term health consequences. Previous research has found that food insecurity was connected to adverse health outcomes (3), higher BMI (5), lower diet quality (5), and general reports of poor health (8, 9). Within the interviews, students highlighted how time constraints, the cost of food, and resource offerings all impacted the quality of foods that they ate and they perceived these foods as “less healthy” as a result.

And finally, the psychological domain captures the ongoing anxiety around navigating food access challenges and aligns with previous research findings (10). In addition to potentially impacting the student's overall health and wellbeing, the stress and anxiety associated with access to adequate food may negatively impact academic performance and success. This finding is supported by previous research that has found food insecurity negatively influences academic performance (3, 4), mental health (7, 8), and physical health (8, 9).

Limitations of the current study include selection bias due to students' voluntary election to be contacted for continued involvement in the authors' larger food security analysis project. Findings were also subject to social desirability bias in participant responses that minimized their severity of food insecurity or need for assistance. Lastly, the 10-item AFSSM with 2-item screener is not a validated tool for food security assessment among college students. Despite these limitations, the authors found notable key themes to demonstrate how college students experience a variety of unique challenges related to different aspects of food access.

### 4.1 Implications

Using a food security screening tool that captures college students' holistic experience can help to proactively address and reduce the negative outcomes associated with food insecurity. These results support the need to further investigate capturing the nuances of food insecurity in college students (33). Researchers have noted limitations with current tools used to measure food insecurity in college students, such as not being formally evaluated for use and accuracy with the college student population and therefore potentially leading to the misclassification of students' food insecurity level (13, 14). As these tools are explored, it is important that the unique experiences of college students, such as those highlighted in this study, are captured by these tools. In addition to capturing whether students are receiving adequate food, there is a need to quantitively capture the additional domains related to social, quality, and psychological experiences.

## 5 Conclusion

The findings of this study suggest that including a complimentary screening tool that captures the social, psychological, and qualitative domains of food insecurity along with the more quantitively focused 10-item AFSSM measurement tool aids in holistically capturing students' experiences. This holistic representation can help better assist colleges in addressing students' needs related to securing and having reliable access to healthy food choices. As a result, this may help in supporting student success, reducing social stigmas related to food assistance, and shaping the policies related to resource use. In addition to qualitative research, future quantitative studies are needed to test the validity and reliability of holistic measurement tools, such as the 4D-FIS (25) and those being developed by The Center for Nutrition and Health Impact (23, 24, for use amongst college student populations.

## Data Availability

The raw data supporting the conclusions of this article will be made available by the authors, without undue reservation.
